# MEG-SCANS - A comprehensive magnetoencephalography speech dataset with Stories, Chirps and Noisy Sentences

**DOI:** 10.1038/s41597-025-06397-4

**Published:** 2025-12-09

**Authors:** Till Habersetzer, Svea Steuer, Andreas Radeloff, Bernd T. Meyer

**Affiliations:** 1 Communication Acoustics, Department of Medical Physics and Acoustics and Cluster of Excellence ”Hearing4all”, Carl von Ossietzky Universität Oldenburg, Oldenburg, Germany; 2 TUD Dresden University of Technology, Dresden, Germany; 3 Department of Otolaryngology, Head and Neck Surgery, Carl von Ossietzky Universität Oldenburg, Oldenburg, Germany

**Keywords:** Databases, Cortex

## Abstract

Stories, Chirps, And Noisy Sentences (MEG-SCANS) is a dataset that provides raw magnetoencephalography (MEG) recordings from 24 German-speaking subjects. Each subject listened to approximately one hour of stimuli, including two audiobooks, sentences from the Oldenburger Matrix Sentence Test (OLSA) for Speech Reception Threshold (SRT) assessment, and short up-chirps used to assess MEG signal quality. The dataset includes MEG data, structural MRI (Magnetic Resonance Imaging) scans of the head, corresponding audio material (audiobooks, OLSA envelopes, and chirp stimuli), and behavioral audiogram and SRT results from hearing screenings. Organized according to the Brain Imaging Data Structure (BIDS), this resource offers a robust benchmark for large-scale encoding/decoding analyses of temporally-resolved brain responses to speech. Comprehensive Matlab and Python code are included to replicate key data validations, ensuring transparency and reproducibility.

## Background & Summary

Understanding how the human brain processes complex, natural speech is a central challenge in neuroscience. While humans effortlessly comprehend highly variable acoustic signals, the precise neurophysiological mechanisms and neuroanatomical infrastructure involved in mapping these signals to symbolic-cognitive units such as phonemes, syllables, and words remain largely unknown^1^. It is hypothesized that robust speech perception arises from a hierarchical auditory processing system that progressively extracts more complex features from the acoustic input^2–7^.

Investigating this hierarchy requires advanced neuroimaging techniques^8,9^. Magnetoencephalography (MEG) is a non-invasive neuroimaging modality with high temporal resolution, making it particularly well-suited for studying regional and large-scale brain dynamics underlying cognitive processes involved in the perception of continuous speech^10,11^. Compared to electroencephalography (EEG), MEG offers superior spatial resolution as magnetic fields are less distorted by the skull, allowing for more accurate localization of cortical sources^11,12^. Combined with anatomical Magnetic Resonance Imaging (MRI), MEG enables precise source-space based analysis, facilitating the reconstruction of neural activity within specific brain regions during speech processing^13–15^. Despite these capabilities, the limited availability of MEG facilities necessitates data sharing to enhance accessibility and to broaden the scientific community’s engagement. Traditional auditory research often relied on short, isolated stimuli to compute auditory evoked potentials or fields (AEP/AEF) to investigate how humans respond to auditory stimuli^16,17^. However, understanding real-world language processing requires analyzing more ecologically valid e.g. naturalistic, continuous speech signals, such as narratives or audiobooks. This poses significant analytical challenges, including the difficulty of mapping temporally variable linguistic units to precise neural signals, disentangling the brain’s response to linguistic features from the highly correlated acoustic features of speech, and overcoming the low signal-to-noise ratio of non-invasive recordings^18–21^. A key approach to address this is the use of linear mathematical models to establish a mapping between specific properties of the speech signal (e.g. the envelope, spectrogram, phonetic features) and the neural response signals^8,9,22,23^. Encoding models map stimulus features to neural signals, while decoding models map stimulus features from neural signals. Using a reconstruction approach, for example by comparing the presented and reconstructed stimulus features, provides a quantitative measure of stimulus encoding in neural signals and allows for investigation into how different features are represented in the brain^8,24,25^.

The dataset presented in this paper aims to fill several gaps in the field: (i) The growing interest in decoding speech directly from brain recordings, particularly with deep learning techniques, highlights a critical need for publicly available and suitable datasets. Currently, there is a gap in open-access data, especially for non-invasive MEG recordings related to continuous speech. A brief, non-exhaustive overview of exemplary MEG datasets for passive listening tasks is presented in Table 1. Our dataset aims to address this gap by providing an open-access resource in the standardized BIDS format for large-scale analyses, building upon efforts to pool data across multiple datasets^26,27^. (ii) The dataset also provides access to speech material presented as speech-in-noise stimuli modulated for varying intelligibility, i.e., the Oldenburger Matrix Sentence Test (OLSA)^28,29^. This data is combined with the behaviorally measured speech reception threshold (SRT), i.e., the signal-to-noise ratio (SNR) at which 50% of words are correctly identified by a listener. This combination enables a direct investigation of the link between neurophysiological responses and behavioral measures of speech comprehension. (iii) It contributes significantly to the existing landscape of publicly available German MEG datasets, as only a limited number of other reported datasets include German language. While Riegel *et al*.^30–32^ reported a dataset with two competing audiobooks, our dataset focuses on a single speaker. (iv) The database uniquely offers data to differentiate between intra-subject effects of acoustic properties across distinct stimulus types: short transient non-speech stimuli (chirps), complex meaningful speech (audiobooks), and meaningless noisy sentences with a simple structure (OLSA).

**Table 1:** Exemplary MEG datasets for passive listening task.

Dataset	Language	Data
van Essen *et al*.^70^ 1200 Subjects Data Release (03/01/2017)	English	95 subjects listening to 7 min brief audio stories
Schoffelen *et al*.^71^	Dutch	102 subjects listening to 120 normal sentences and 120 scrambled sentences
Armeni *et al*.^15^	English	3 subjects listening to 10 h of audiobooks
Wang *et al*.^72^	Mandarin Chinese	12 subjects listening to 6 h of audio stories
Gwilliams *et al*.^73^	English	27 subjects listening to 2 h of audio stories
d’Ascoli *et al*.^26^	French	58 subjects listening to 100 min of an audiobook; data unavailable at time of writing
Riegel *et al*.^30–32^	German	58 subjects listening to 40 min of competing audiobooks
Li *et al*.^74^	Chinese	30 subjects listening to 25 min of multispeaker video using a 64-channel optically pumped magnetometer (OPM) MEG system
Özdogan *et al*.^75^	English	1 subject listening to 50 h of audiobooks; data not yet accessible

To address these points, the open-access MEG-SCANS dataset provides MEG data from 24 German-speaking subjects. Each subject contributed approximately 40 min of audiobook recordings, 120 sentences in noise from the OLSA matrix test ( ≈10 min per participant), and 240 short chirp stimuli, unless stated otherwise. This extensive collection yielded approximately 16 h of MEG data from the audiobook recordings and 4 h from the OLSA sentences.

## Methods

### Subjects

Twenty-four adults (16 females, 8 males; mean age = 25 years, SD = 3), all native speakers of German, were recruited from the University Campus Oldenburg. Prior to their participation, all subjects were screened for normal hearing using a pure-tone audiogram and reported no history of neurological disorders. Normal hearing was defined as a four-frequency (0.5, 1, 2, 4 kHz) pure-tone average (4fPTA) below 20 dB HL^33^. Subjects provided written informed consent for participation in the study and for the open sharing of their anonymized data and were compensated for their time. Handedness was not formally assessed or reported for this study. Subjects sub-01 and sub-02 completed a preliminary version of the experimental paradigm. All subsequent subjects (sub-03 onwards) underwent the final version of the paradigm. The study was approved by the University of Oldenburg Ethics Committee (DRS.EK/2019/034).

### Procedure

All Magnetoencephalography (MEG) measurements were conducted using an Elekta Neuromag Triux system. This system is equipped with 306 sensors, comprising 204 planar gradiometers and 102 magnetometers. These sensors are arranged into 102 triplets, each consisting of one magnetometer and two orthogonal planar gradiometers. Measurements were performed with subjects in an upright position (68^∘^). MEG signals were recorded at a sampling frequency of 1000 Hz and online band-pass filtered between 0.1 Hz and 330 Hz.

#### Study Design

**Fig. 1:**
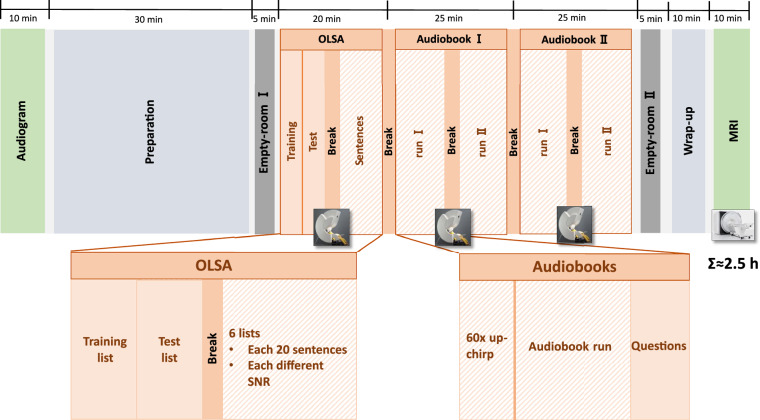
Schematic of the experimental paradigm, detailing the sequence and content of the MEG measurement blocks.

The experimental paradigm is illustrated in Fig. 1. The order of experimental blocks remained constant across all subjects. Prior to the MEG measurements, the pure-tone audiogram was measured using an Auritec T900 audiometer and Sennheiser HDA 200 headphones within a sound-attenuated booth. Each subject underwent seven distinct MEG recordings, interspersed with brief breaks. During the experiment, subjects were instructed to maintain their gaze on a fixation cross displayed on the screen.

- **task-noise:** For noise estimation, two empty-room MEG recordings, each lasting approximately three minutes, were acquired at the beginning and end of the experiment.
- **task-olsa:** 120 German sentences from the Oldenburger Matrix Sentence Test^28^ were presented. The sentences, spoken by a male speaker, were divided into six lists, each containing 20 sentences, and embedded within a continuous background noise stream. This noise was generated from the speech material itself, ensuring it shared the same long-term frequency spectrum as the sum of all sentences. Each list was presented at a fixed estimated target intelligibility level (20%, 40%, 50%, 60%, 80% and 95%). The corresponding SNRs required to achieve these intelligibility levels were estimated based on each subject’s fitted psychometric function^34^, described by:
  $$p(x)=\frac{1}{1+{e}^{-4m(x-{L}_{50})}}$$
  Here, *p*(*x*) is the probability of correct recognition at a given SNR *x*. The slope *m* was fixed at 0.15 for all subjects according to Brand *et al*.^34^. The SRT, denoted as *L*_50_, was individually estimated for each subject prior to the main sentence presentation. *L*_50_ corresponds to the SNR at which a subject achieves a 50% probability of correct responses. This estimation employed an adaptive procedure using two lists of 20 sentences each (one for training, one for testing), conducted within the MEG. The subjects were instructed to repeat the words they understood from each sentence. This adaptive procedure continuously adjusted the speech presentation level *L* to converge on each subject’s *L*_50_. Sentences during the following OLSA task were presented with an inter-stimulus interval (ISI) of at least 1 s, with an additional 200 ms jitter. Sentence presentation order was randomized, and individual sentence onsets were precisely triggered.
- **task-audiobook:** Two German public-domain audiobooks were presented, each approximately 20 min in duration. These audiobooks were narrated by members of the working group: one male speaker for each audiobook. The stories selected were both by Edgar Allan Poe: “The Tell-Tale Heart” and “The Masque of the Red Death”. Each audiobook was divided into two parts for presentation. After each audiobook part (i.e., after each block), subjects answered three comprehension questions. The audiobooks were continuously triggered every second, aligned with the audio stream. At the beginning of each audiobook block, prior to the audiobook playback, 60 short frequency sweeps (up-chirps from 0.1 Hz to 10 kHz)^35,36^ were presented with an ISI of at least 1.1 s, plus 200 ms jitter. These up-chirps served as short transient stimuli, providing a well-understood baseline measurement to assess MEG signal quality.

**Acoustic stimulation** was delivered diotically using foam tips via Tip-300 insert earphones, driven by an RME Fireface UCX soundcard (RME, Haimhausen, Germany) and a TDT HB7 headphone driver (Tucker-Davis, Alchua, FL, United States). Trigger signals were generated simultaneously as an aligned audio channel from the soundcard, transmitted as a digital signal to a custom-made trigger box, and subsequently to the MEG system. This custom-built FPGA-based trigger box utilized the clock of the Fireface UCX to convert the SPDIF output into a 5 V TTL trigger input to the MEG. All audio signals were presented at a sampling rate of 44100 Hz. The background noise in the OLSA task was maintained at 65 dB SPL, as was the long-term sound level of the two audiobooks. Chirps were presented at a comfortable listening level.

**A T1-weighted anatomical magnetic resonance imaging (MRI) scan** of the head was acquired for each subject at the end of the MEG experiment or during an additional appointment. Imaging was performed using a Siemens Magnetom Prisma 3T scanner. Structural MRIs were collected using a 3D MPRAGE sequence, yielding 0.75 mm isotropic resolution images (FOV = 240 mm, matrix = 320 × 320 × 240, sagittal slices). Key sequence parameters included: TR = 2000 ms, TE = 2.07 ms, TI = 952 ms, FA = 9^°^, and a pixel bandwidth of 250 Hz. A GRAPPA acceleration factor of 2 was applied for in-plane parallel reduction, with 0.1 phase encoding oversampling. To maintain subject anonymity, the T1-weighted MRI anatomical scans were defaced during BIDS conversion using MNE-BIDS^37^.

**Subject preparation** before each MEG session involved the application of three additional bipolar electrode channels to monitor physiological artifacts: one electrocardiogram (ECG) channel and two electrooculography (EOG) channels (vertical, VEOG, and horizontal, HEOG) for eye movements. Five head position indicator (HPI) coils were attached to each subject head using a custom-made cap. The anatomical fiducial points, the HPI coil positions, and the head shape were then digitized with a hand-held Polhemus Fasttrak device. Head position was continuously monitored throughout MEG recordings.

#### Computing environment

Beyond the specific packages highlighted in this manuscript, the processing of the presented data relied on the free and open-source software ecosystem. The following tools, in particular, were utilized for BIDS dataset preparation, the subsequent analysis and forced alignment of audio files with their orthographic transcription:

- MNE-BIDS^37,38^ (https://mne.tools/mne-bids/stable/index.html)
- MNE-Python^39,40^ (https://mne.tools/stable/index.html)
- Bids-Validator (https://bids-standard.github.io/bids-validator/)
- FreeSurfer^41^ (https://surfer.nmr.mgh.harvard.edu)
- FieldTrip^42^ (https://www.fieldtriptoolbox.org)
- mTRF-Toolbox^9^ (https://github.com/mickcrosse/mTRF-Toolbox)
- Auditory Modelling Toolbox^43^ (https://amtoolbox.org)
- Montreal Forced Aligner^44^ (https://montreal-forced-aligner.readthedocs.io)

## Data Records

**Fig. 2:**
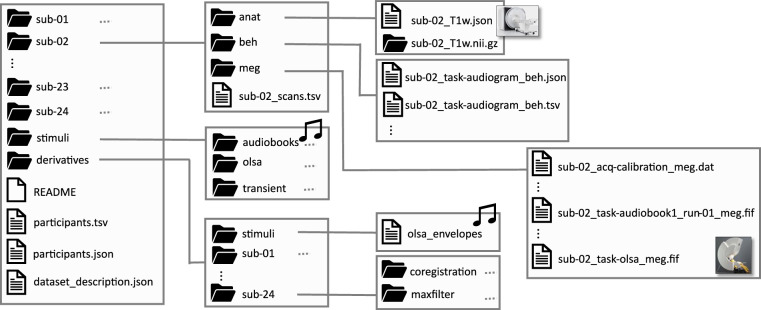
The BIDS data structure for the MEG-SCANS dataset. The folder for an example sub-02 is expanded in more detail, alongside key contents of the stimuli and derivatives directories.

The MEG-SCANS dataset is publicly available on the OpenNeuro platform^45,46^ under a Creative Commons CCO license. It is organized according to the Brain Imaging Data Structure (BIDS) version 1.10.0^47,48^, and the dataset can be accessed at https://openneuro.org/datasets/ds006468/versions/1.1.1. A partial depiction of the BIDS folder structure is provided in Fig. 2. For a comprehensive understanding of the BIDS file system, please refer to the official specification at https://bids.neuroimaging.io. In total, the dataset includes recordings from 24 subjects, each contributing a single session. The root directory of the dataset contains the following files:

- *dataset_description.json*: Provides general metadata about the dataset.
- *participants.tsv*: Contains demographic information (age and gender) for each subject.

### Stimuli

Additionally, a dedicated *stimuli* directory contains the materials for the three stimulus types: up-chirps, audiobooks and OLSA sentences.

- A *.wav*-file (*upchirp_stim.wav*) of the chirp stimulus presented at the beginning of the audiobook recordings.
- The four audiobook recordings (*.wav*), each accompanied by its orthograhic transcription (*.txt*) and a time-aligned TextGrid file with word and phone boundaries.
- Background noise and sentences used during the OLSA recording. Since the OLSA sentences are part of a commercial product, the sentences and the noise derived from these cannot be provided. Instead, the corresponding envelopes are included to enable replication of stimulus properties without revealing the actual sentences. The corresponding orthographic transcriptions (*.txt*) and time-aligned TextGrids are available.

For each subject, the dataset provides data across three primary modalities: **MEG** measurements, **anat**omical recordings, and **beh**avioral data. Any exceptions to complete data availability are detailed in the “Subject Data Integrity and Exceptions” subsection below.

### MEG Recordings

The MEG recordings consist of seven files per subject:

- **Empty-room recordings [task-noise]:** Two empty room measurements were conducted for noise estimation: *run-01* (before the subject’s session) and *run-02* (after the subject’s session).
- **Audiobook recordings [task-audiobook]:** Four audiobook recordings correspond to two distinct stories, each divided into two runs (*audiobook1_run-01*, *audiobook1_run-02*, *audiobook2_run-01*, *audiobook2_run-02*).
- **Oldenburg Matrix Sentence Test (OLSA) recording [task-olsa]:** A block containing 120 sentences from the OLSA, presented at six different signal-to-noise ratios (SNR) with 20 sentences presented at each SNR.

### Anatomical Recordings

The anatomical data primarily consists of a T1-weighted structural MRI scan. This MRI has been coregistered to the MEG coordinate frame, specifically the *task-audiobook1_run-01* recording.

### Behavioral Data

The behavioral data for each subject includes:

- **Pure-tone audiogram [task-audiogram]:** Documents hearing thresholds for the left and right ear.
- **OLSA Matrix Test [task-OlsaMatrixTest]:** Contains measured SRTs for the OLSA obtained during both training and the main test run. The latter SRT was specifically used during the OLSA MEG task to individualize stimulus presentation at target intelligibility levels.
- **Intelligibility [task-intelligibility]:** Based on the measured SRT and a predetermined psychometric slope, a psychometric function was fitted (Eq. (1) in^34^). This allowed for the computation of individualized SNRs corresponding to predefined intelligibility levels.
- **Audiobook comprehension questions [task-AudiobookQuestions]:** After each of the four audiobook recordings, three comprehension questions were posed. The resulting data represent the percentage of correct answers.

### Derived Data

The dataset also includes processed data for each subject, located within the *derivatives* directory:

- **MaxFilter:** Data preprocessed using MNE-Python’s implementation of Maxwell filter^49,50^. This preprocessing includes spatiotemporal signal space separation (tSSS), correction for head movements, and transformation to a common head position across all subject recordings (relative to *task-audiobook1_run-01*) to facilitate coregistration. A comprehensive HTML report from this stage provides estimated head positions, Power Spectral Densities (PSDs) before and after filtering, and the 3D head position transformations applied for inter-run alignment.
- **Coregistration:** Provides the transformation matrices used for MEG-MRI coregistration. This has been calculated by MNE-Python using the subject’s headshape. Cortical reconstructions, generated by FreeSurfer’s *recon-all* pipeline, were used for this step but are withheld from public sharing due to privacy concerns regarding subject identifiability.
- **OLSA envelopes**: Due to sharing restrictions, the original OLSA *wav-files* cannot be shared. Instead, only the envelopes utilized during the Technical Validation are provided. Other derived features from the OLSA sentences are available upon request.

### Subject Data Integrity and Exceptions

The final experimental paradigm was consistently applied to 22 of the 24 subjects (sub-03 onwards) in this dataset. Subjects sub-01 and sub-02 completed a preliminary version with minor deviations from this final paradigm.

- **sub-01:** This subject served as a pilot for a preliminary experimental paradigm. This included simultaneous MEG and EEG (32 channels), differing from the main cohort’s MEG-only measurements. Preliminary versions of the audiobooks used for sub-01, which are slightly modified compared to the final versions, are also provided in the *stimuli* directory specific to this subject (*pilot*). Furthermore, chirp stimuli were not included in sub-01’s audiobook recordings, and the *audiobook2* recordings have been withheld due to distribution restrictions on the source audio.
- **sub-02:** For sub-02, the final audiobooks were used. However, the experimental paradigm for the chirp stimuli within the audiobook recordings involved 150 chirp presentations per block instead of the standard 60 used for all subsequent subjects. This modification exclusively affects the chirp stimulus and does not impact the recorded MEG signals of the audiobooks themselves.
- **sub-11:** The *task-noise_run-02* recording is missing.

## Technical Validation

Dataset compliance with the Brain Imaging Data Structure was verified using the BIDS-Validator version 2.2.0. To ensure data quality, several well-established analysis approaches were employed. Auditory evoked fields (AEFs) were computed from responses to chirp stimuli and analyzed with dipole fitting, serving as a reference to confirm measurable auditory responses and the general applicability of source modeling methods^16,51,52^. Furthermore, two methods were applied to establish a systematic relationship between continuous natural speech and brain responses. First, a cross-correlation analysis between MEG signals and speech onset envelopes was conducted^53^. Second, a linear decoding model for envelope reconstruction was trained and tested on audiobook data^20,54,55^. This trained decoder was then applied to unseen OLSA sentences to demonstrate a link between behaviorally measured speech intelligibility and MEG signals^56^. Given its robustness for stimulus reconstruction with this type of data^57^, our approach uses a linear decoder that capitalizes on the cortical entrainment to the speech envelope which focuses on the low-frequency range^58,59^. We implemented this using a backward linear model, often called a temporal response function (TRF), which reconstructs the speech envelope from the multivariate neural recordings. For this purpose, we utilize the widely-adopted mTRF-Toolbox for MATLAB^9^. The performance of such decoding models is typically quantified using Pearson’s or Spearman’s correlation (*r*) to measure the similarity between the original and the reconstructed stimulus feature.

All MEG data were preprocessed using the MaxFilter as described previously. Apart from the application of MaxFilter, preprocessing was kept minimal. Recorded EOG and ECG data were not utilized for artifact removal (e.g., eye blinks or heartbeats). Unless otherwise specified, all analyses were performed on data from all 24 subjects.

### Chirps

#### Signal Processing

The chirp stimuli were analyzed for subjects sub-02 to sub-24. The data was analyzed in both sensor space and source space. In sensor space, auditory evoked fields were computed by pooling all 240 chirp stimuli (600 for sub-02) across all runs. To this end, the data were bandpass filtered between 1 and 40 Hz and epoched from −0.5 s to 0.5 s relative to stimulus onset. An artifact rejection procedure was then applied, discarding epochs where the signal amplitude’s Z-score exceeded an empirically determined threshold (>6). The remaining epochs were then baseline corrected and averaged for each subject. Subsequently, the group grand-average AEFs were calculated across all subjects. Using the BIDS-dataset’s coregistration information, coregistered head and source models were created within FieldTrip. For each subject, a dipole fit was performed separately for magnetometers and gradiometers within the 90 ms to 130 ms time window of the AEFs encompassing the N100m-component. Following the individual subject fits, the grand-average dipole positions and grand-average dipole moments were calculated across subjects. Subjects with poor fits (e.g., unilateral dipoles or extreme outlier dipoles) were excluded from the grand-average.

#### Results

**Fig. 3:**
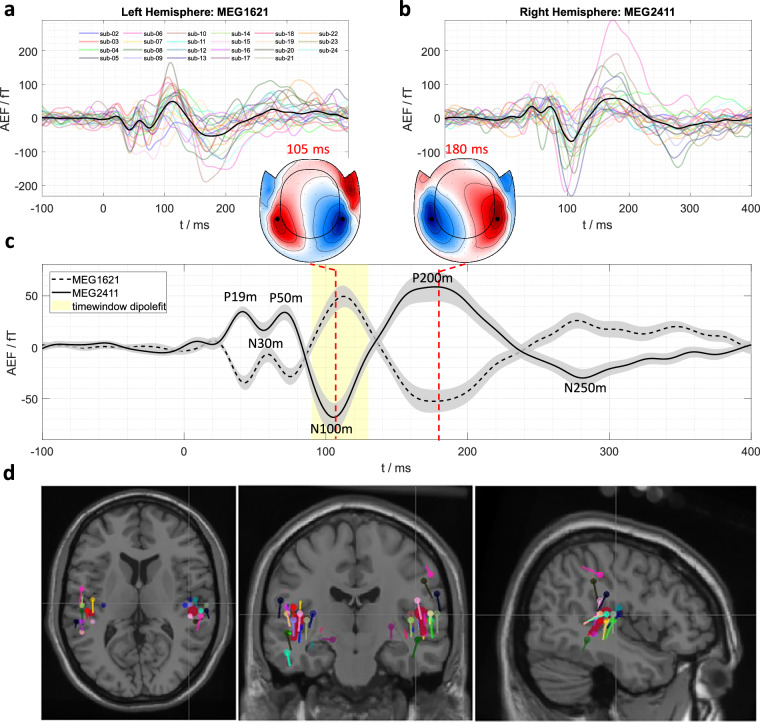
(**a,b**) Auditory evoked fields for the left and right hemispheres, exemplified by two magnetometer channels MEG1261 and MEG2411. Black lines show the grand-average AEFs, while individual subject AEFs are color-coded (see legend). (**c**) Grand-average AEFs (from a,b) with standard error of the mean (SEM). Topographic maps of the N100m and P200m components are depicted. The locations of the two exemplified magnetometers are marked with black dots on the maps. The time window for the N100m component dipole fit is highlighted by the shaded yellow region. (**d**) Individual subject (color-coded) and grand-average (thick dark red) two-dipole model fits derived from gradiometer data, presented in axial, coronal, and sagittal views. The crosshair marks the right Heschl’s gyrus. The Colin 27 average brain served as the stereotaxic template.

Figure 3 presents the results from both the sensor-level and source-level analyses. The auditory evoked fields exhibit typical waveforms and topographical field maps consistent with auditory activity in MEG, displaying clear dipolar patterns in both hemispheres^17,60^. Grand-average dipoles were localized in the superior temporal gyrus (STG) near Heschl’s gyrus (HG)^61,62^. While individual dipole localizations showed some variability, this demonstrates the general applicability of source modeling methods. More elaborate artifact rejection techniques could potentially improve the localization of individual fits.

### Cross-correlation analysis

#### Signal Processing

For the cross-correlation analysis, continuous MEG data from all audiobook recordings were bandpass filtered (0.5 Hz to 45 Hz), segmented into consecutive 10 s epochs, and downsampled to 250 Hz. Subsequently, all preprocessed audiobook MEG trials from all runs were concatenated. Speech onset envelopes were extracted from audiobooks by first calculating the absolute value of the analytical signal, which was derived via the Hilbert Transform. This signal was then low-pass filtered at 25 Hz, its first derivative was taken, it was half-wave rectified, and finally downsampled to 250 Hz. Normalized cross-correlation functions were computed between the derived onset envelope and each MEG channel, using a time window from  − 100 ms to 900 ms (where a positive lag indicates the audio signal preceding the MEG response). As a control condition, cross-correlation functions were also calculated using a random (shuffled) permutation of audio epochs. For both the original and shuffled conditions, a mean cross-correlation function was computed for each subject, followed by the calculation of grand-average cross-correlation functions across all subjects. To identify statistically significant differences between the sorted and shuffled conditions, we performed a non-parametric, cluster-based permutation test across all magnetometers^63^. This non-parametric approach effectively controls for the multiple comparisons problem. The significance of the resulting clusters was determined using 1000 permutations, with a final alpha level of p  < 0.025. Our analysis focused on clusters showing sustained significance within two primary time windows of interest: 50 ms to 90 ms and 160 ms to 230 ms ms.

#### Results

**Fig. 4:**
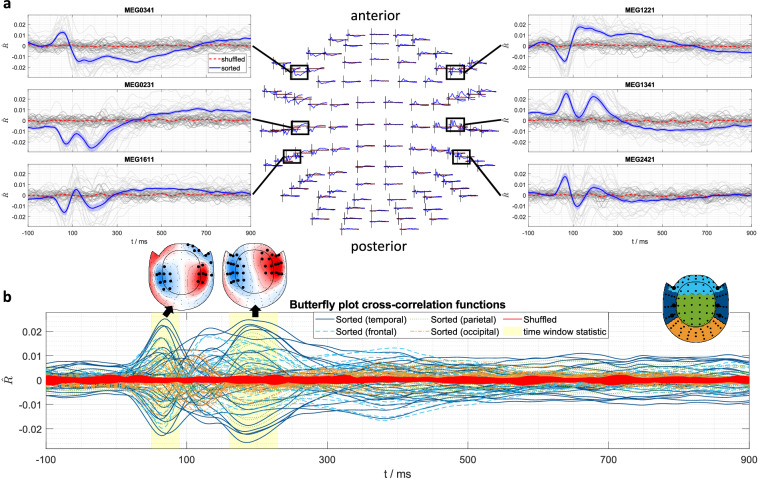
(**a**) Topographical representation of cross-correlation functions between continuous audiobook speech onset envelopes and MEG magnetometer signals, spanning latencies from  − 100 ms to 900 ms. The sorted audio condition is presented in blue, while the shuffled condition is shown in red. Additionally, zoomed-in panels illustrate the time courses from six specific sensors, depicting individual subject data in light grey for the sorted condition and darker grey for the shuffled condition. (**b**) The central butterfly plot shows the waveforms for the sorted condition, color-coded by anatomical region, and for the shuffled condition in red. An inset topographical map clarifies the color-coded sensor groupings. Two topographical plots display the mean difference in correlation values (sorted minus shuffled) for two key time windows. Statistically significant sensor clusters throughout the entire time window, identified by a cluster-based permutation test on the contrast between conditions, are highlighted as black dots. Corresponding gradiometer data are available but not included in this figure.

Figure 4 presents the results of the cross-correlation analysis. The cross-correlation functions show curves similar in magnitude and morphology to previously reported findings^53^. As expected, the shuffled audio control condition is flat, indicating no systematic relationship between the audio and cortical response, in contrast to the sorted audio. The cross-correlation functions for the sorted audio display expected patterns at characteristic latencies within the first few hundred milliseconds, primarily in temporal areas known to be involved in auditory processing^14,58^. This observed neural activity was statistically validated by the cluster-based permutation test, which located significant clusters in temporal areas that encompassed the primary peaks of the cross-correlation function.

### Decoding analysis

#### Signal Processing

The decoding analysis aimed to predict speech envelopes using a linear decoder. The decoder was further used to predict unseen sentences from the Oldenburger Matrix Sentence Test. Based on the findings of Vanthornhout *et al*.^56^ that the delta band is optimal for decoding continuous speech used in hearing tests, the continuous MEG data from the audiobooks was bandpass filtered between 0.5 Hz and 4 Hz, segmented into consecutive 120 s epochs, and downsampled to 64 Hz. Data from all audiobook runs were combined, and all trials were retained. For the 120 OLSA sentences, MEG data for each subject were epoched from sentence onset (0 ms) to sentence duration + 0.5 s, also bandpass filtered (0.5 Hz to 4 Hz), and downsampled to 64 Hz. For audio data preprocessing, speech envelopes for both OLSA and audiobook stimuli were extracted according to Biesmans *et al*.^64^. This involved using a Gammatone filterbank with 28 channels (each covering one equivalent rectangular bandwidth, ERB, with center frequencies from 5 Hz to 5000 Hz). The absolute value of each channel’s output was calculated and raised to the power of 0.6 to derive the envelope, and these envelopes were then averaged. The averaged envelope was subsequently filtered with the same settings as the MEG signal (0.5 Hz to 4 Hz) and downsampled to 64 Hz. For normalization, both MEG signals and speech envelopes were normalized via Z-score. To preserve spatial MEG patterns, the Z-score for MEG data was calculated based on all combined magnetometer or gradiometer channels, and these common normalization values were applied uniformly to the respective channels. For decoder training and application, an individual linear decoder was trained for each subject using audiobook recordings. All trials were first randomly shuffled and then partitioned into a training set (80% of the data) and a validation set (the remaining 20%). The regularization parameter was determined via cross-validation on the training set. The trained decoder’s performance was verified on the held-out validation data under both sorted and shuffled audio conditions. This decoder utilized all latencies between 0 ms and 250 ms for envelope reconstruction^65^. Finally, the trained decoder was applied to the unseen OLSA sentences. For each sentence, varying in SNR during the experiment, the Spearman correlation between the envelope prediction and the original envelope was calculated.

#### Results

**Fig. 5:**
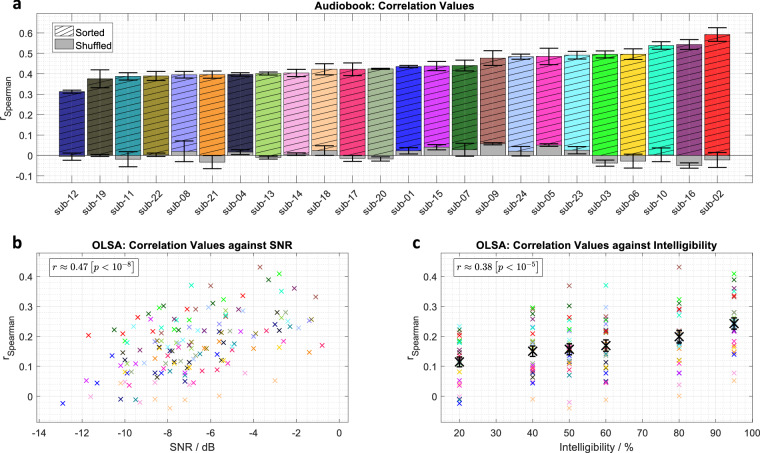
(**a**) Individual subject correlation values for audiobook data between the envelope prediction and both the original audio and a shuffled version on the held-out test set, including standard errors of the mean. Each subject has a consistent color coding across all subfigures. (**b**) Individual subject correlation values for predicted versus original envelopes for each OLSA sentence at given SNRs. (**c**) Individual subject correlation values for predicted versus original envelopes for each OLSA sentence at given intelligibility levels. For both (**b**) and (**c**), each data point represents the average of 20 OLSA sentences. Fixed intelligibility levels across subjects resulted in individual SNRs for the sentences. Spearman correlation values between the depicted correlation values and SNR or intelligibility were found to be significant.

Figure 5 presents the results of the decoding analysis. For sorted audiobook test data, correlation values are higher than for shuffled data, which centers around zero. The grand-average correlation for the sorted audiobook condition (*M* = 0.443, *SD* = 0.013) was significantly higher than for the shuffled audiobook condition (*M* = 0.0033, *SD* = 0.0057), as confirmed by a dependent samples t-test (*t*(23) = 30.02, *p* < . 001). This indicates that the decoders are capable of predicting the speech envelope, demonstrating a systematic relationship between MEG and the audio envelope for each subject. The correlation values fell within the expected range, even slightly exceeding typical results from EEG, which reach up to 0.35^66^. This improvement is attributed to the greater number of sensors that MEG employs for stimulus reconstruction. Concerning the OLSA sentences, reconstruction accuracies, as measured by correlation values, were significantly correlated with both the presented SNRs and intelligibility. This pattern reveals an expected gradual increase in reconstruction accuracy as both SNR and intelligibility improve in line with previously reported values^56,67,68^.

## Data Availability

The MEG-SCANS dataset is publicly available on the OpenNeuro platform under a Creative Commons CCO license and can be accessed at https://openneuro.org/datasets/ds006468/versions/1.1.1^46^.

## Code availability

The code for the Technical Validation, including a detailed description, is available on GitHub under the BSD 3-Clause License (https://github.com/tillhabersetzer/MEG-SCANS)^69^. The provided code demonstrates the practical usage of the database files and reproduces all figures presented in the Technical Validation section.
